# The impacts of math anxiety, science anxiety, and gender on arts *versus* sciences choices in Qatari secondary schools

**DOI:** 10.7717/peerj.14510

**Published:** 2023-01-09

**Authors:** Ahmed M. Megreya, Ahmed A. Al-Emadi

**Affiliations:** Department of Psychological Sciences, College of Education, Qatar University, Doha, Qatar

**Keywords:** Math anxiety, Science anxiety, STEM careers, Gender differences

## Abstract

Previous studies showed small-to-moderate associations between students’ performances in math and science and math anxiety and science anxiety, respectively. Accordingly, the high prevalence of these two forms of topic anxiety represent severe obstructions to the worldwide demand calling for improving the quality of math and science achievements and, subsequently, increasing career success in science, technology, engineering, and math (STEM) domains. Therefore, this study examined math anxiety and science anxiety among female and male students who were enrolled in Sciences *vs* Arts tracks in Grades 11 and 12 in a Middle Eastern Arabic-speaking country (Qatar), and investigated how gender, math anxiety and science anxiety could predict this enrollment. Results showed that students in the Arts track experienced higher levels of math anxiety and science anxiety than those in the Sciences track, regardless of the students’ gender. However, a binary logistic regression analysis showed that science learning anxiety, but not evaluation science anxiety nor math learning or evaluation anxieties, significantly predicts students’ enrollment in Arts and Sciences tracks. Therefore, STEM career success is associated with good knowledge of STEM domains *and* positive emotions towards math and science.

## Introduction

Recent research from a range of interdisciplinary scientific fields, including educational psychology, clinical psychology, education, and cognitive neuroscience, have provided cumulative evidence that emotions greatly influence students’ learning and achievement (for extensive reviews, see Pekrun & Linnenbrink-Garcia, 2014; Pekrun & Loderer, 2020). For example, these emotions showed significant impacts on students’ attention, motivation, learning strategies, and self-regulation (Pekrun, 2014). Specifically, Pekrun (2014) identified four groups of what were termed academic emotions, including achievement emotions (*e.g*., fear of failure), epistemic emotions activated by cognitive difficulties (*e.g*., frustration), topic-related emotions (*e.g*., math anxiety), and social emotions (*e.g*., social anxiety). Interestingly, topic-related emotions, especially math anxiety and science anxiety, are the most investigated forms of academic emotions as they are commonly considered as barriers to success in the domain of science, technology, engineering, and mathematics (STEM; Beilock & Maloney, 2015; Carey et al., 2016; Foley et al., 2017; Mallow, 2006).

### Math anxiety

Math anxiety refers to the feelings of fear, tension, and apprehension upon exposure to math-related materials during learning and assessment (Ashcraft, 2002). In a recent meta-analysis, Barroso et al. (2021) found a small to moderate negative correlation between math anxiety and math achievement. However, other studies found that math anxiety was not a predictor of math achievement (Abín et al., 2020). Rather, Wang et al. (2018) found that high exam math anxiety was present in students with all levels of math motivation (high, median, and low), whereas higher learning math anxiety was generally associated with lower math motivation. Accordingly, Wang et al. (2018) suggested that highly math-anxious students are not *always* unmotivated in math.

Importantly, however, evidence indicates that highly math-anxious students tend to avoid math as much as they could (Moustafa, Porter & Megreya, 2020; Ramirez, Shaw & Maloney, 2018). For example, a meta-analysis reported that highly math-anxious students took fewer high school mathematics courses and showed less intention in high school and college to take more mathematics (Hembree, 1990). In addition, highly math-anxious students tend to avoid STEM domains in universities by avoiding majors that had moderate or high math requirements (Beilock & Maloney, 2015; Foley et al., 2017; LeFevre, Kulak & Heymans, 1992).

Furthermore, longitudinal studies have confirmed the role of math anxiety on STEM domains and careers avoidance (Ahmed, 2018; Daker et al., 2021). For example, Ahmed (2018) examined the contributions of math anxiety in predicting later STEM career choices in a large sample of seventh grade students who were followed for 7 years. Specifically, Ahmed (2018) distinguished four heterogeneous math anxiety trajectory groups: consistently low, decreasing, increasing, and consistently high. Adolescents in the consistently low math anxiety group were about 7.4 times as likely to be employed in STEM-related professions compared to the consistently high math anxiety adolescents. In addition, adolescents in the decreasing math anxiety group were 6.4 times as likely to be employed in STEM-related professions as those in the consistently high math anxiety group. In another longitudinal study, Daker et al. (2021) measured math anxiety and math ability in a sample of first-semester university students whose STEM avoidance and achievement were tracked through their transcripts during 4 years. Daker et al. (2021) found that math anxiety data that were collected during the students’ first-semester prospectively predicted their real-world university STEM participation and achievement, even when individual differences in math ability were controlled. Therefore, Daker et al. (2021) concluded that math anxiety represents a barrier to STEM participation and achievement greater than math ability itself.

Gender was also found to be a predictor of math anxiety, as female students experience math anxiety higher than male students (Van Mier, Schleepen & Van den Berg, 2018; Xie et al., 2019), even when math performance and test anxiety were controlled (Chinn, 2009; Devine et al., 2012; Dowker, Sarkar & Looi, 2016; Else-Quest, Hyde & Linn, 2010). This gender difference has been attributed to math-related gender stereotypes, where math is traditionally viewed as a male domain (Plante, Theoret & Favreau, 2009). Therefore, math anxiety was thought to be responsible for the finding that far fewer women than men take part in high school and college mathematic courses (Hembree, 1990).

### Science anxiety

Science anxiety is defined as fears and tensions that can occur before or during science learning (Bryant et al., 2013; Mallow et al., 2010). Therefore, highly science-anxious students tend to avoid science and STEM domains (Mallow, 1994). For example, science anxiety was positively associated with choosing university majors in humanities and social sciences (Mallow, 1994; Udo et al., 2001, 2004). In addition, increased science anxiety was found to relate with lower levels of science achievement (Britner & Pajares, 2006; Kupermintz, 2002; Pajares, Britner & Valiante, 2000), SAT-Quantitative scores (Brownlow, Jacobi & Rogers, 2000), science vocabulary knowledge (Ardasheva et al., 2018), and self-efficacy (Griggs et al., 2013; Ardasheva et al., 2018; Britner, 2008).

Female students experience higher science anxiety than male students (Ardasheva et al., 2018; Britner, 2008; Mallow, 1994; Mallow et al., 2010; Megreya, Szűcs & Moustafa, 2021). Consistently, previous studies showed that females carry more negative attitudes toward science than male students (George, 2006). In addition, females were found to achieve lower than males in physics (Taasoobshirazi & Carr, 2008), even when they outperform males in terms of GPA (Hofer & Stern, 2016). These gender differences might be due, at least in part, to gender-related stereotyping that considers science as a male domain (Mallow, 2006; Rahm & Charbonneau, 1997).

### Current study

Previous studies have separately examined the contribution of math anxiety and science anxiety to the avoidance of STEM domains (*e.g*., Ahmed, 2018; Daker et al., 2021; Mallow, 1994; Udo et al., 2001, 2004). No study has previously compared the contributions of these two forms of topic anxiety. Fortunately, the constructs of math anxiety and science anxiety can be measured using two structurally related questionnaires: the modified Abbreviated Math Anxiety Scale (m-AMAS; Carey et al., 2017) and the Abbreviated Science Anxiety Scale (ASAS; Megreya, Szűcs & Moustafa, 2021).

The m-AMAS was adapted for use with children from the Abbreviated Math Anxiety Scale (AMAS; Hopko et al., 2003), which measures how anxious students would feel during certain situations in math class. Exploratory and confirmatory factor analyses consistently reported a two-factor structure for the AMAS (Hopko et al., 2003) that was robustly replicated for the m-AMAS (Carey et al., 2017). These factors are learning math anxiety (*i.e*., the feelings of tension induced during math learning activities) and math evaluation anxiety (*i.e*., the feelings of tension associated with taking assignments and tests in math). The ASAS (Megreya, Szűcs & Moustafa, 2021) is adapted from the m-AMAS (Carey et al., 2017) to measure science anxiety in children and adolescents by replacing “math” environments with “science” contexts. Confirmatory factor analysis supported the two-factor structure of the ASAS (learning science anxiety and science evaluation anxiety; Megreya, Szűcs & Moustafa, 2021).

The present study aimed to investigate math anxiety, science anxiety, and gender as predictors of students’ choices of Sciences and Arts tracks in secondary schools in Qatar. Based on preferences, during the end of the second semester of grade 10, secondary school students in most, if not all, Arab countries are divided into two main tracks: Sciences (which include advanced chemistry, physics, biology, and math) and Arts (which include history, geography, psychology, sociology, and philosophy). More information about this educational system can be found in UNESCO’s Global Education Monitoring Report (UNESCO, 2019) in Arab countries.

## Method

### Participants

A total of 344 students in grades 11 and 12 in two government secondary schools (one for females and one for males) in Qatar volunteered to participate in this study. This sample represented 51.9% of the student population in grades 11 and 12 in those two schools. A power analysis supported the generalizability of the sample size. The G*Power software (Faul et al., 2007) gave an actual power of 0.95, with an effect size ≥0.25 and a sample size of 280. All participants were asked to report their gender (giving 173 females and 171 males), age (with a mean of 18.9 years and 7 months SD). Table 1 shows the main descriptions of this sample. Data were collected with approval from the Ministry of Education and Higher Education in Qatar. In addition, eithical approval was obtained from Qatar University’s IRB committee (QU-IRB 1356-EA/20), which required a written consent approval from all participants.

**Table 1:** Characteristics of participants.

		Grade 11		Grade 12	
		Sciences	Arts	Sciences	Arts
Females	*N*	46	42	41	45
	Age (mean and SD in years)	16 (1)	16.4 (0.7)	16.9 (0.4)	17.1 (0.6)
Males	*N*	45	36	40	49
	Age (mean and SD in years)	16.5 (0.7)	17.1 (1.1)	17.3 (0.7)	17.8 (0.8)

### Instruments

(1) *The Modified-Abbreviated Math Anxiety Scale: Arabic translation (m-AMAS- Ar.:* Carey et al., 2017). The m-AMAS (Carey et al., 2017) was adapted from the AMAS (Hopko et al., 2003) to be more suitable for British children. It consists of nine items measuring Learning Math Anxiety (LMA; five items) and Math Evaluation Anxiety (MEA (four items)). In addition, a total score represents the summation of these two factors. Participants respond to each item using a 5-point Likert ranking scale, ranging from one (low anxiety) to five (high anxiety).

(2) *The Abbreviated Science Anxiety Scale* (*ASAS*; Megreya, Szűcs & Moustafa, 2021). The ASAS has been adapted from the m-AMAS (Carey et al., 2017) to measure science anxiety using a 5-point Likert ranking scale, ranging from one (low anxiety) to five (high anxiety). The ASAS consists of nine items, which belong to two main factors: LSA (five items) and SEA (four items). In addition, a total score represents the summation of these two factors.

## Procedure

During the first 3 weeks of the first semester in grades 11 and 12, the questionnaires were administered in groups throughout class attendance with fixed order of scales (the m-AMAS then the ASAS) across all participants. Adminstration lasted for approximately 10 min and all participants were encouraged to respond and not skip any item.

### Statistical analyses

All statistical analyses were conducted using IBM SPSS Version 27.0 (IBM Corp, Armonk, NY, USA), except McDonald’s Omega coefficient (ω), which was conducted using JASP Version 0.16.3 (JASP Team, 2022). Using the whole sample, reliability and correlations were examined. McDonald’s ω was used to examine the internal reliability of the m-AMAS and the ASAS. Pearson correlation coefficients were used to examine the inter-correlations between the sub-scales of each questionnaire and the correlations between math and science anxiety. In order to examine the differences between females/males in Sciences/Arts tracks in grades 11/12, a series of 2 × 2 × 2 between-participant Analyses of Variance (ANOVAs) was conducted for math anxiety and science anxiety separately. A Binary Logistic Regression Analysis was conducted to examine the impact of math anxiety, science anxiety, and gender on Arts and Sciences choices (which were coded using 0 for Arts and 1 for Sciences). The Value of Wald test and the ROC curve were used to examine predictability. Finally, we report means, 95% confidence intervals, standard errors and standard deviations.

## Results

### Internal reliability

Table 2 shows McDonald’s Omega coefficient (ω) of the scales, with 95% confidence Intervals (CIs) in parenthesis. Good to adequate reliabilities were observed for the math and science anxiety scores across the four groups of students that ranged from 0.78 to 0.93 (for the total score), from 0.64 to 0.90 (for the learning subscales), and from 0.74 to 0.89 (for the evaluation subscales).

**Table 2:** McDonald’s Omega coefficient (ω) (95% CI) of math and science anxiety scales.

	Arts	Sciences
Math anxiety	0.88 [0.85–0.91]	0.92 [0.90–0.94]
Learning math anxiety	0.81 [0.77–0.86]	0.87 [0.85–0.90]
Math evaluation anxiety	0.80 [0.75–0.85]	0.88 [0.85–0.91]
Science anxiety	0.79 [0.75–0.84]	0.83 [0.80–0.87]
Learning science anxiety	0.75 [0.69–0.81]	0.73 [0.67–0.79]
Science evaluation anxiety	0.84 [0.80–0.88]	0.84 [0.80–0.88]

### Inter-correlations of math and science anxiety subscales

Positive inter-correlations were observed between learning *vs*. evaluation anxiety subscales for the m-AMAS-Ar, *r* (342) = 0.74, *p* ≤ 0.001 ([0.69–0.79], 95% CI) and the ASAS, *r* (342) = 0.49, *p* ≤ 0.001 ([0.40–0.57], 95% CI).

### Correlations between math and science anxiety

There were strong positive correlations between math and science anxiety for the total score, *r* (342) = 0.54, *p* ≤ 0.001 ([0.46–0.61], 95% CI) and the two subscales: learning anxiety, *r* (342) = 0.51, *p* ≤ 0.001 ([0.43–0.58], 95% CI) and evaluation anxiety *r* (342) = 0.55, *p* ≤ 0.001 ([0.47–0.62], 95% CI).

### Differences between sciences and arts tracks

**Figure 1:**
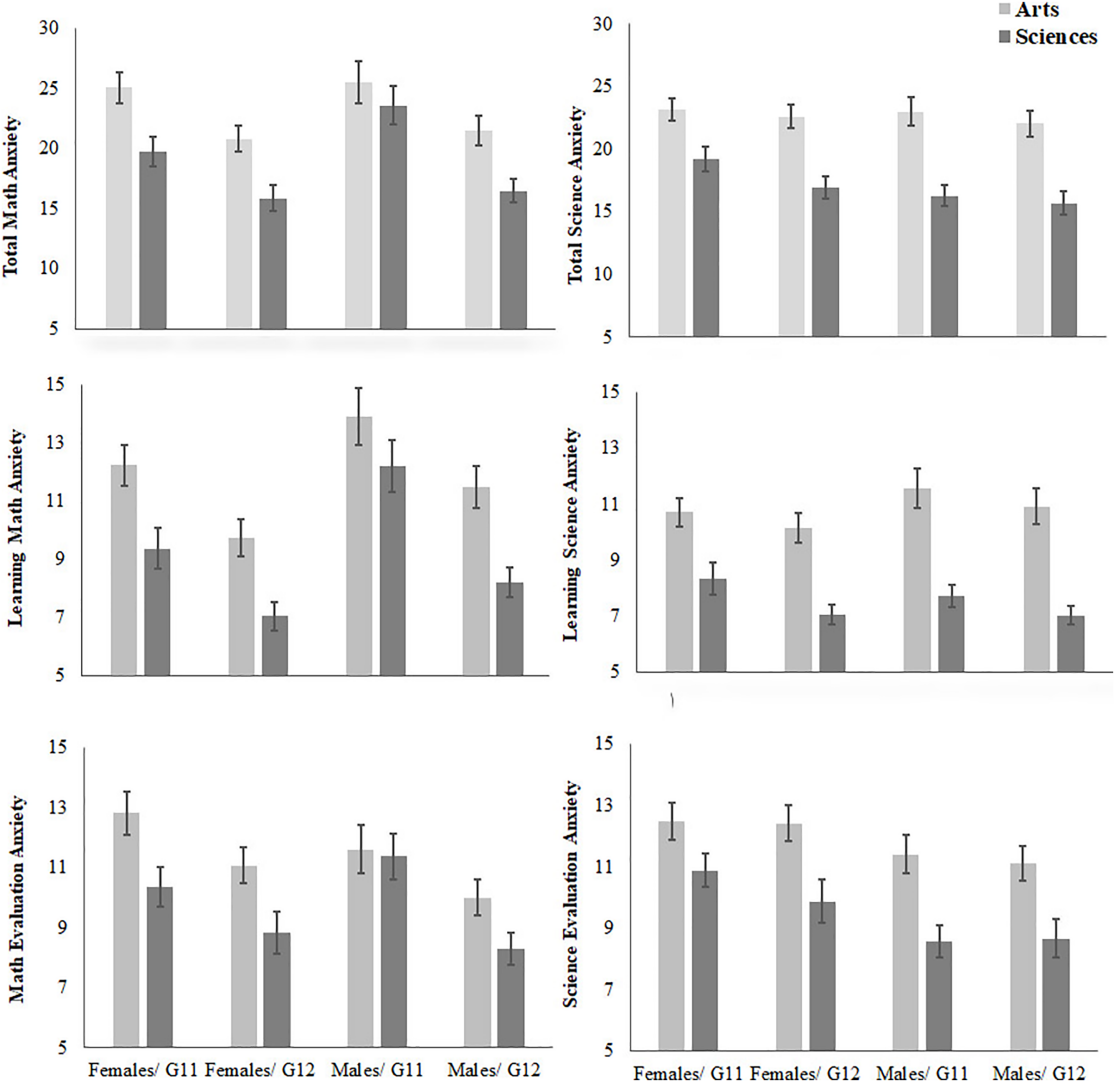
The differences between females/males in Arts/Science tracks in science/math anxiety in Grades 11 and 12.

Figure 1 presents descriptive statistics of math anxiety and science anxiety for female and male students in Sciences and Arts tracks in Grades 11 and 12. For science anxiety, 2 × 2 × 2 between-participant ANOVAs showed main effects of Tracks for the total score, *F* (1, 336) = 68.78, *p* ≤ 0.001 (means of 22.7 *vs* 17.1), LSA, *F* (1, 336) = 78.29, *p* ≤ 0.001 (means of 10.8 *vs* 7.6), and SEA, *F* (1, 336) = 30.56, *p* ≤ 0.001 (means of 11.8 *vs* 9.5, all for Arts and Sciences; respectively). In addition, there was a main effect of Gender for SEA, *F* (1, 336) = 11.91, *p* ≤ 0.001 (means of 11.4 *vs* 9.9, for females and males; respectively). Furthermore, there was a main effect of Grade for LSA, *F* (1, 336) = 4.41, *p* = 0.04 (means of 9.4 *vs* 8.9, for Grade 11 and Grade 12; respectively). No other main effects nor interactions were found, *F* (1, 336) ≤ 1.

For math anxiety, similar analyses revealed main effects of Tracks for the total score, *F* (1, 336) = 21.51, *p* ≤ 0.001 (means of 23 *vs* 19.1), LSA, *F* (1, 336) = 26.38, *p* ≤ 0.001 (means of 11.7 *vs* 9.3), and SEA, *F* (1, 336) = 11.99, *p* ≤ 0.001 (means of 11.3 *vs* 9.8, all for Arts and Sciences; respectively). In addition, there were main effects of Gender for MLA, *F* (1, 336) = 13.01, *p* ≤ 0.001 (means of 11.4 *vs* 9.6, for females and males; respectively). Furthermore, there were main effects of Grade for the total score *F* (1, 336) = 26.83, *p* ≤ 0.001 (means of 23.3 *vs* 18.9), LSA, *F* (1, 336) = 29.96, *p* ≤ 0.001 (means of 11.8 *vs* 9.2), and SEA, *F* (1, 336) = 17.23, *p* ≤ 0.001 (means of 11.5 *vs* 9.6, all for Grade 11 and Grade 12; respectively). No other main effects nor interactions were found, *F* (1, 336) ≤ 1.

### Arts *vs* sciences choices

**Figure 2:**
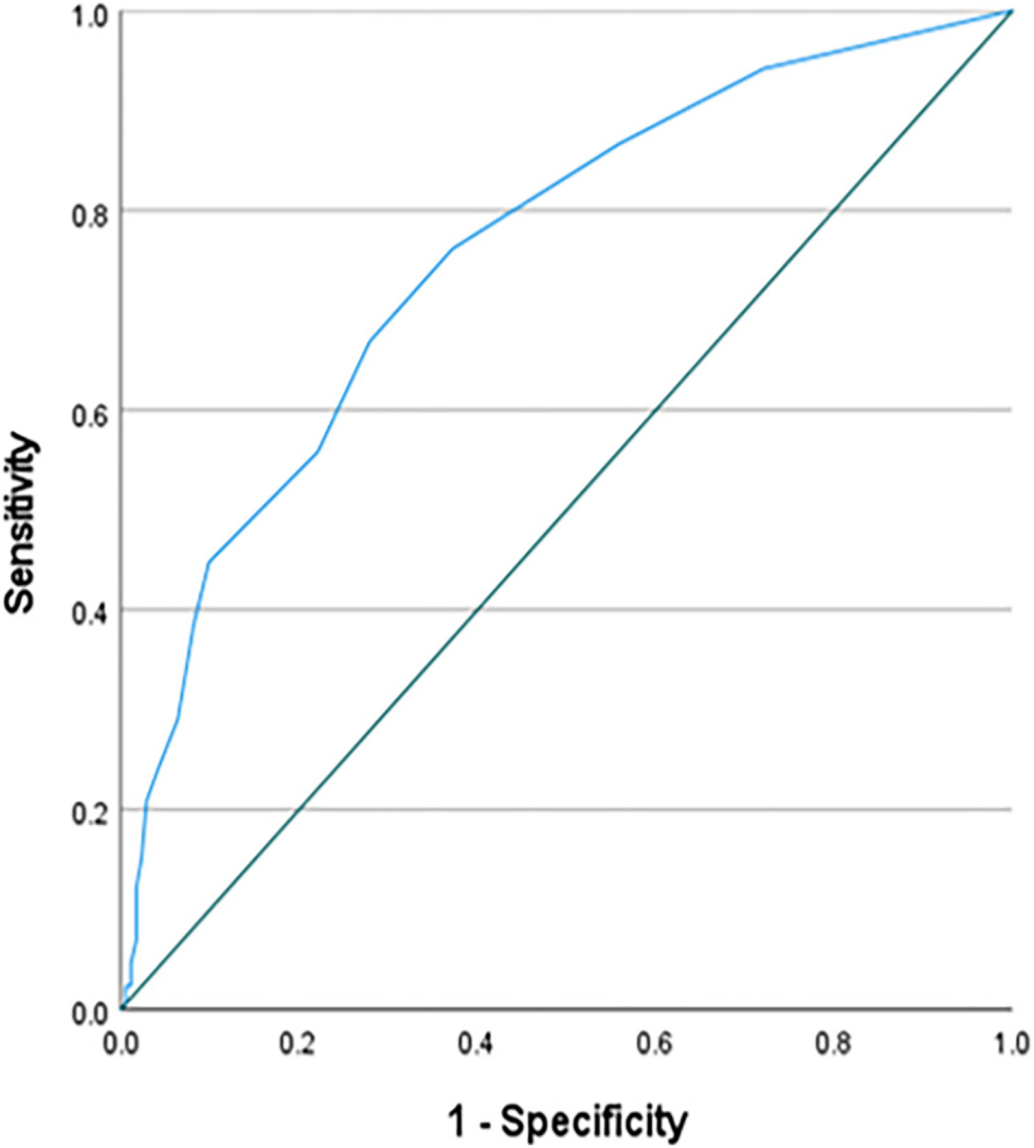
The ROC curve for learning science anxiety.

The model of a Binary Logistic Regression Analysis was significant, *X*^*2*^ (5) = 72.532, *p* ≤ 0.001, with an overall classification percentage of 68.3. However, as presented in Table 3, only science learning anxiety was a significant predictor (Value of Wald test = 24.879, *p* = ≤ 0.001 Exp(B) = 0.78). Figure 2 presents the ROC curve for science learning anxiety, where the area under the curve was 0.757 ([0.707–0.808], 95% CI), SE = 0.026, *p* ≤ 0.001. The coordinates of the ROC curve reported a cut-off point of 9.5 or higher that predicts Arts track with = 76.2% sensitivity. The value under the cut-point predicts Sciences track with specificity 63%.

**Table 3:** Comparisons among students groups in math anxiety and science anxiety.

	Wald	*p*
Gender	0.947	0.330
Science learning anxiety	24.879	0.000
Science evaluation anxiety	2.020	0.155
Math learning anxiety	0.333	0.564
Math evaluation anxiety	0.441	0.507

## Discussion

The main aim of the present study was to examine the differences in math anxiety and science anxiety among female and male students who chose enrollment in Sciences *vs* Arts tracks in secondary schools in a Middle Eastern Arabic-speaking country (Qatar). In addition, the predictability of students’ choices was investigated using these two forms of topic anxiety along with gender. The results showed that students in Arts track experience higher levels of math anxiety and science anxiety than those in Sciences track, regardless of their gender. Binary Logistic Regression Analysis showed that science learning anxiety, but not evaluation science anxiety nor math anxiety subscales, significantly predicted the students’ choices in Sciences *vs* Arts tracks.

It is well established that students’ performances in math and science negatively correlate with math anxiety and science anxiety, respectively (*e.g*., for reviews see Carey et al., 2016; Britner, 2010; Megreya, Szűcs & Moustafa, 2021). Accordingly, it has been suggested that high prevalence of these two forms of topic anxiety (*e.g*., for reviews see Dowker, Sarkar & Looi, 2016; Mallow, 2006) represent a severe obstruction for the worldwide demand to improve the quality of math and science achievements and subsequently, to increase STEM career success (Beilock & Maloney, 2015; Foley et al., 2017).

Indeed, many studies have consistently found that math anxiety (Chipman, Krantz & Silver, 1992; Beilock & Maloney, 2015; Foley et al., 2017) and science anxiety (Mallow, 1994; Udo et al., 2001, 2004) associated negatively with interest in scientific careers in university students. Similar results were reported for high school students (*e.g*., Espino et al., 2017; Megreya, Szűcs & Moustafa, 2021). For example, math anxiety was associated with choosing humanities and social sciences among students in grade 11 in Philippines (Espino et al., 2017). In addition, our previous study found that students who enrolled in the Arts track in Grades 11 and 12 experience higher levels of science anxiety than those in Sciences track (Megreya, Szűcs & Moustafa, 2021). Consistently, the present study found that students in Arts track experience higher levels of both science anxiety and math anxiety than those in Sciences track. More importantly, science-learning anxiety was the sole predictor the students’ choices in Sciences *vs* Arts tracks. This finding may be a reflection of the study plan of students in the Sciences track, where science courses represent more than one third of the total score whereas there is only one course for math.

In fact, the causal relationship between science/math anxiety and the choices of Arts *vs* Sciences tracks is not clear. From one hand, the higher levels of science and math anxiety might contribute for not choosing the Sciences track. This possibility could be supported by the previous findings that math anxiety represents a barrier to STEM participation and achievement among university students (*e.g*., Daker et al., 2021). From the other hand, studying sciences and math in the Sciences track could also be a factor for decreasing the levels of science and math anxiety. Supporting this possibility, students in Grade 12 showed lower levels of math and science learning anxiety than those in Grade 11. This finding suggests that the recurrent and organized confrontation of math- and science-related materials could reduce math and science learning anxiety. Consistently, extensive sessions of tutoring led to a significant reduction of math anxiety (*e.g*., Supekar et al., 2015). Enhanced academic performance could be a mediating factor for the effects of tutoring on math anxiety. However, repeated and systemic exposure of math-related materials could also reduce math anxiety (Dowker, Sarkar & Looi, 2016). Consistently, a large body of clinical studies supported the effectiveness of exposure-based treatments for anxiety-related disorders (*e.g*., for reviews see Foa & McLean (2016); Moscovitch, Antony & Swinson (2009)). These results, therefore, might provide a new avenue for treating topic-related learning anxiety.

Interestingly, the present study showed for the first time strong positive correlations between math anxiety and science anxiety. Previous studies reported that math anxiety shares similar features with other forms of anxiety such as general anxiety and test anxiety (Lauer, Esposito & Bauer, 2018). However, no previous studies have examined a joint factor structure between math anxiety and science anxiety. Intuitively, Henschel (2021) suggested that math anxiety and science anxiety might share similar features. Therefore future studies need to investigate whether math anxiety and science anxiety could be involved in a single concept or represent distinct constructs.

To conclude, as math anxiety and science anxiety negatively correlate with students’ self-efficacy in math and science (*e.g*., Griggs et al., 2013), students with high levels of these two forms of anxiety tend to avoid STEM courses. These negative emotions toward math and science might be causes *or* effects for the lower performances in these topics, representing a significant barrier to STEM career success. Accordingly, efficient intervention programs for math/ science anxiety could be a key factor for increasing STEM career participation and success, which are associated with good knowledge of STEM domains *and* positive emotions towards math and science.

## Additional Information and Declarations

### Data Availability

The following information was supplied regarding data availability: The raw measurements are available in the Supplemental Files.
